# Chiral Thianthrenes

**DOI:** 10.3390/ijms25084311

**Published:** 2024-04-13

**Authors:** M. John Plater, William T. A. Harrison

**Affiliations:** Department of Chemistry, University of Aberdeen, Meston Walk, Aberdeen AB24 3UE, UK

**Keywords:** thianthrene, dithiin, sulfoxide, chiral sulfoxide, configuration, diastereoisomer, resolution

## Abstract

The absolute configuration and stability of two thianthrene chiral sulfoxides has been determined by means of X-ray single-crystal structure determinations. The analyses and configurations allow verification that the diastereomeric sulfoxides are stable in solution and are not interconverting, which has been suggested in some studies of sulfoxides. The two thianthrene sulfoxides have slightly different *R*_f_ values, which allowed their separation using flash chromatography on silica. The spots run back-to-back, which posed a challenge for their separation. The pure, separated compounds in solution remain as separate, single spots on a Thin Layer Chromatography (TLC) plate.

## 1. Introduction

Stereogenic and configurationally stable sulfur atoms, such as sulfoxides and sulfinate esters, have been known for a century [1,2,3]. The earliest examples of optically active forms of sulfoxides were reported in 1926 [4,5] and by 1962, a method for making enantio-enriched isomers was known [6,7,8]. From this time, chiral sulfoxides have been of interest in asymmetric synthesis [9,10,11,12] and drugs [13,14]. Numerous reviews are available which cover their synthesis and properties [15,16,17,18,19,20,21]. Notable is the instability of some chiral sulfoxides, which rearrange into racemic sulfoxides and make correct enantiopurity analysis difficult [22,23,24,25,26,27,28,29]. Kagan initially reported this observation [23], but it was not accepted widely until later, making many observations on enantiopurity in the literature doubtful [15]. Racemic thianthrene sulfoxides are rare but some are known such as 1-methylthianthrene-5-oxide [30] and 4-methylthianthrene-5-oxide [30], 2,7-dimethylthianthrene-5-oxide [31], 1-*p*-tolylsulfanylthianthrene-10-oxide [32] and chlorpromazine-S-oxide [33]. Asymmetric sulfide oxidation is useful for forming chiral sulfoxides [34,35,36,37,38]. Thianthrene racemates with adjacent chiral centres have been formed by the addition of thianthrene cation radical salts to cycloalkenes and alkenes [39]. Also, regiospecific alkene amino functionalisation was achieved via an electrogenerated dielectrophile [40]. Our previous heterocyclic synthesis studies with 1,2-dinitro-4,5-difluorobenzene led to X-ray single-crystal determinations of phenazines, ref. [41] phenoxazines and phenothiazines [42,43]. Heterocyclic syntheses with this building block have been expanded here to explore the X-Ray single-crystal structures and stabilities of chiral, enantiomerically pure, thianthrene sulfoxides.

## 2. Results and Discussion

**Figure 1:**
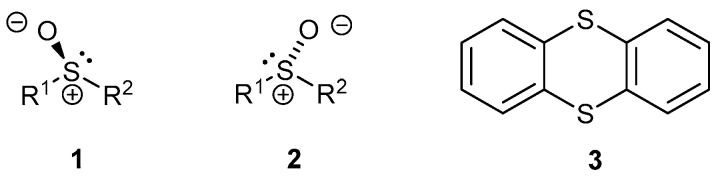
Drawings of chiral sulfoxides and thianthrene.

In this work, enantiopure sulfoxides of the thianthrene framework **3** are prepared for the first time. This has a folded butterfly shape that is expected to invert rapidly, but we anticipated that making a sulfoxide from one of the sulfur atoms would fix the conformation. Attaching a chiral centre would give two diastereoisomers with different physical and chemical properties, allowing them to be separated and characterised, provided that the sulfoxide is stable enough to hold its configuration. The configuration of a chiral sulfur atom can be assigned using a sub-rule of the Cahn–Ingold–Prelog (CIP) sequence rules [44,45]. The CIP rules are based on quadri-covalent asymmetric atoms, and a lone pair of electrons, on nitrogen or sulfur, has negligible mass. An imaginary or phantom atom, of low priority, is added on here. A configuration of either *R* or *S* can then be assigned (Figure 1). 

**Figure 2:**
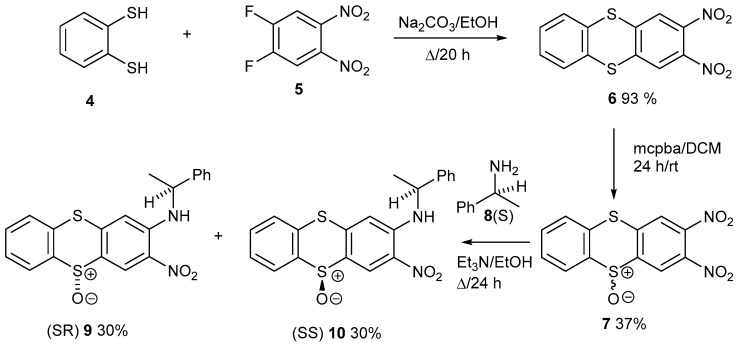
Synthesis of enantiomerically pure thianthrenes **9** and **10**.

**Figure 3:**
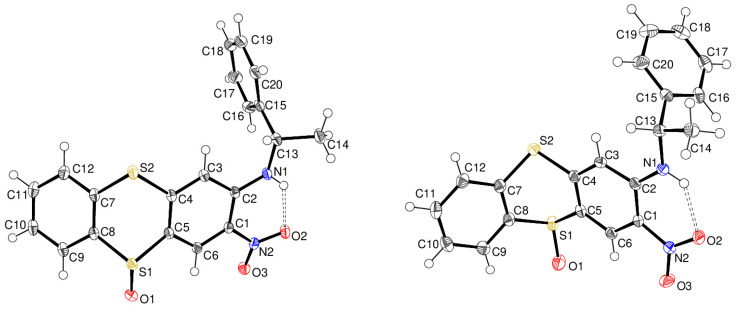
The molecular structures of **9** (**left**) and **10** (**right**) showing 50% displacement ellipsoids. The hydrogen bonds are shown as double-dashed lines. The sulfoxides orientate in the direction of the thianthrene puckering. Oxygen is red, blue is nitrogen and yellow is sulfur.

These are the first thianthrenes prepared using 1,2-difluoro-4,5-dinitrobenzene, **5**. The reaction of benzenedithiol **4** with compound **5** and Na_2_CO_3_ gave 2,3-dinitrothianthrene **6** (Figure 2). Oxidation with *meta*-chloroperbenzoic (mcpba) acid gave an enantiomeric mixture of sulfoxides **7**. The oxidation of the second sulfur atom was not observed, suggesting that one sulfoxide deactivates the other sulfur atom. Treatment with (*S*)-phenylethylamine **8** displaced the 3-nitro group, which is conjugated to the electron-withdrawing sulfoxide group. The two nitro groups activate each other, but the sulfoxide provides additional activation to the 3-nitro group. Only products **9** and **10** were formed in equal amounts, which is reasonable given their similar framework. They eluted as two back-to-back spots on a TLC plate with an eluent of dichloromethane/ether (90:10). Using chromatography on flash silica, they were resolved and separated, and then characterised using NMR and X-Ray single-crystal structure determinations. The proton and ^13^C NMR data are virtually identical, making it impossible to distinguish the two diastereoisomers. Figure 3 shows the molecular structures of both diastereoisomers **9** and **10** side by side. The yields for each step are shown in Figure 2 by the side of the compound number. The yields are calculated as the ratio of the moles of dry product divided by the moles of starting material x 100 to convert this into a percentage. The maximum possible yield is 100%. A yield of 70–80% is very good and over 90% is excellent and very high. Yields of 37% and 30% are much lower and are workable over a short scheme, but a great deal of mass is lost, which makes it difficult to carry on with further steps. For example, 500 mg of compound **6** gave 191 mg of sulfoxide **7**, a 37% yield. This work was challenging because of two low yields, the back-to-back TLC spots and split material. The remaining material in each reaction had no products which were easily isolated. It was mainly baseline and intractable. Compounds **6**–**7** and **9**–**10** are crystalline solids, which dried easily in air, but compound **11**, an orange/red oil, had DCM in the proton and 13 carbon NMR. This evaporated after a beaker of it was left exposed to the atmosphere for two months and the spectra re-run.

The products were characterised using X-Ray single-crystal structure determinations. A guide to crystallography is available [46]. Compound **9** (Figure 3) crystallises in the trigonal space group *R*3 (No. 146). The dihedral angle between the C1–C6 and C7–C12 rings is 43.56 (5)°, resulting in a ‘butterfly’ conformation for the fused ring system; atoms S1, O1 and S2 deviate from the mean plane of C4/C5/C7/C8 by −0.746 (2), −0.142 (4) and −0.560 (2) Å, respectively. As expected, S1 is pyramidal [deviation from C5, C8 and O1 = −0.6872 (9) Å], the S1–O1 bond length is 1.4890 (14) Å, indicating a significant degree of double-bond character, and the C5–S1–C8 bond angle is 97.10 (7)°. These data compare with the corresponding S atom displacement, S–O separation and C–S–C angle of 0.684 (2) Å, 1.489 (6) Å and 96.9 (3)°, respectively, in the centrosymmetric co-crystal of thianthrene 5-oxide with 1,4-di-iodotetrafluorobenzene [47]. The dihedral angle between the C1–C6 ring and the pendant C15–C20 ring in **9** is 85.96 (6)°, and the C2–N1–C13–C14 torsion angle is 154.86 (16)°. An intramolecular N1–H1^…^O2 hydrogen bond [H^…^O = 2.02 (3) Å, N–H^…^O = 133 (2)°] generates an *S*(6) ring. The absolute structure (C13 *S*, S1 *R*) is well established and consistent with the starting materials. No significant directional intermolecular interactions were identified in the extended structure. 

**Figure 4:**
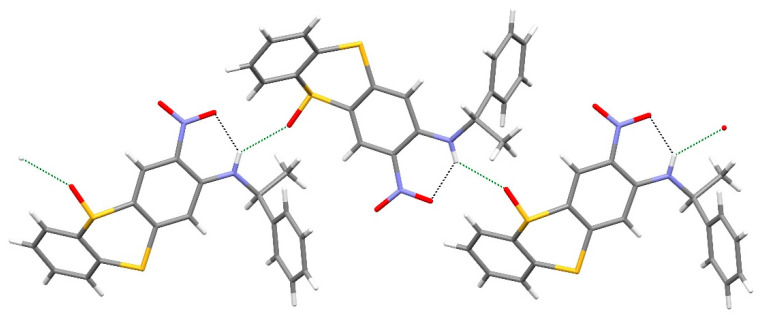
Fragment of a hydrogen-bonded [001] chain of molecules in the extended structure of compound **10**. Note that the NH group participates in both intramolecular and intermolecular links. Oxygen is red, blue is nitrogen and yellow is sulfur..

Compound **10** (Figure 3) crystallises in the orthorhombic space group *P*2_1_2_1_2_1_ (No. 19). The C1–C6 and C7–C12 rings of the thianthrene fused ring system subtend a dihedral angle of 52.55 (5)°, and the equivalent angle between the C1–C6 and C15–C20 rings is 81.47 (6)°. Atoms S1, O1 and S2 deviate from the C4/C5/C7/C8 plane by 0.831 (3), 0.356 (5) and 0.663 (3) Å, respectively. The C2–N1–C13–C14 torsion angle is 160.95 (19)° and an intramolecular N1–H1^…^O2 hydrogen bond [H^…^O = 1.97 (3) Å, N–H^…^O = 134 (2)°] occurs. Unlike compound **9**, the N–H group in compound **10** also forms a weak intermolecular hydrogen bond to the sulfoxide O atom [H^…^O = 2.57 (3) Å, N–H^…^O = 136 (2)°], which results in [001] chains in the extended structure (Figure 4) [48]. The absolute structure is well established with C13 *S* and S1 *S*. 

**Figure 5:**
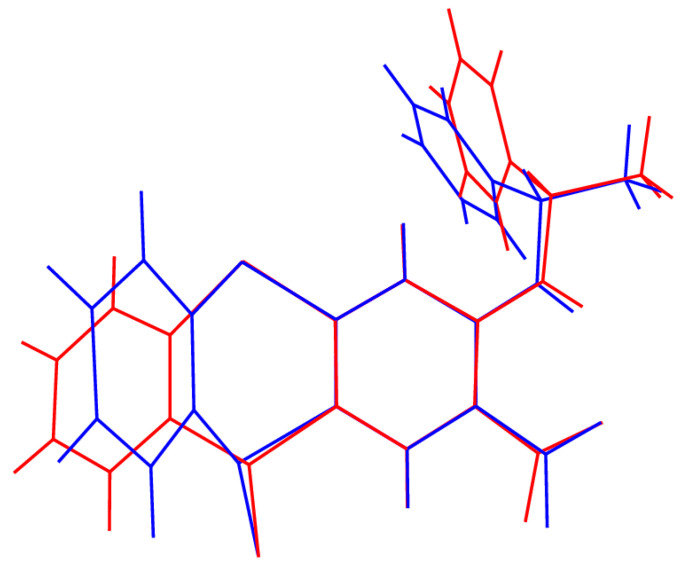
An overlay view of **9** (red) and **10** (blue) showing the different orientations of the terminal aryl ring owing to the rigid sulfoxide chiral centre. Atoms C1–C6 in the two structures are superimposed (the blue ring comes forward and the red ring goes back).

Although the dihedral angles between the aryl rings of the thianthrene ring systems are similar in compounds **9** and **10**, the overall molecular conformations are quite different, as illustrated in an overlay plot [49] (Figure 5), which shows that the C7–C12 ring is ‘flipped’ up or down in the two structures due to the rigid, chiral, sulfoxide moiety. Both structures are well ordered with no suggestion of disorder. 

**Figure 6:**
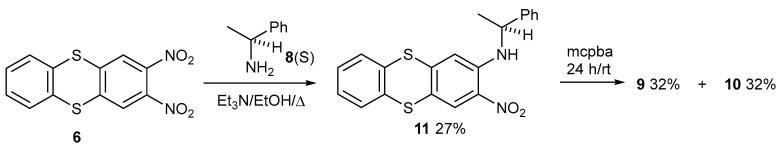
An alternative pathway to thianthrenes **9** and **10**. Supplementary Materials are available from the electronic site below.

Thianthrenes **9** and **10** were also prepared in similar yields from the mcpba oxidation of thianthrene **11** (Figure 6). This was prepared from 2,3-dinitrothianthrene **6** through the displacement of one of the nitro groups. One of the sulfur atoms in compound **11**, conjugated to the amine group, is more electron-rich, so it might have oxidised more readily, but the oxidation was still a low-yielding reaction. Sulfoxides **9** and **10** formed in equal amounts, so the energy pathways to their formation, as two different diastereoisomers, must be similar.

## 3. Materials and Methods

IR spectra were recorded on a diamond-attenuated total reflection (ATR) Fourier transform infrared (FTIR) spectrometer (Thermo Fisher Scientific, Stafford House, Boundary Way, Hemel Hempstead, UK). Ultraviolet (UV) spectra were recorded using an Evolution, UV-Vis spectrometer with EtOH as the solvent (Thermo Fisher Scientific, Stafford House, Boundary Way, Hemel Hempstead, UK). The term sh means shoulder. ^1^H and ^13^C nuclear magnetic resonance (NMR) spectra were recorded at 400 and 100.5 MHz, respectively, using a Bruker 400 spectrometer (Research Complex at Harwell, Rutherford Appleton Laboratory, Harwell, Didcot, Oxon, UK). Chemical shifts, δ, are given in ppm and measured by comparison with the residual solvent. Coupling constants, *J*, are given in Hz. A broad signal is abbreviated as br. High-resolution mass spectra were obtained at the University of Wales, Swansea, using an Atmospheric Solids Analysis Probe (ASAP) (positive mode) instrument: Xevo G2-S ASAP (Waters Corporation, 34 Maple Street, Milford, MA, USA). Melting points were determined on a Cole-Palmer MP-200D Stuart digital melting point apparatus (9 Orion Court, Ambuscade Road, Colmworth Business Park, St Neots, Cambridgeshire, UK). All chemicals were purchased from Sigma-Aldrich, Gillingham, UK.The crystal structures of **9** (yellow plate 0.10 × 0.05 × 0.03 mm) and **10** (yellow slab, 0.12 × 0.10 × 0.03 mm) were established using intensity data collected on a Rigaku CCD diffractometer (Mo Kα radiation, λ = 0.71073 Å for **9** and Cu Kα radiation, λ = 1.54178 Å for **10**) at 100 K. (Malvern Panalytical Ltd., Barn B, 2 Cygnus Business Park, Middle Watch, Swavesey, Cambridge, UK). 

The structures were routinely solved by dual-space methods using SHELXT [50] and the structural models were completed and optimized by refinement against |*F*|^2^ with SHELXL-2018 [51]. The N-bound H atoms were located in difference maps and their positions were freely refined. The C-bound H atoms were placed geometrically (C–H = 0.95–1.00 Å) and refined as riding atoms. The methyl groups were allowed to rotate, but not to tip, to best fit the electron density. The constraint *U*_iso_(H) = 1.2*U*_eq_(carrier) or 1.5*U*_eq_(methyl carrier) was applied in all cases. Full details of the structures and refinements are available in the deposited cifs. 

Crystal data for **9**: C_20_H_16_N_2_O_3_S_2_, *M*_r_ = 396.47, trigonal, space group *R*3 (No. 146), *a* = 20.3224 (4) Å, *c* = 11.3272 (2) Å, *V* = 4051.38 (17) Å^3^, *Z* = 9, *T* = 100 K, Mo Kα radiation, λ = 0.71073 Å, *R*(*F*) = 0.039 [7914 reflections with *I* > 2σ(*I*)], *wR*(*F*^2^) = 0.083 (9333 reflections), Flack absolute structure parameter = 0.04 (2), CCDC deposition number 2337820. 

Crystal data for **10**: C_20_H_16_N_2_O_3_S_2_, *M*_r_ = 396.47, orthorhombic, space group *P*2_1_2_1_2_1_ (No. 19), *a* = 7.97576 (14) Å, *b* = 13.9104 (3) Å, *c* = 16.8489 (3) Å, *V* = 1869.32 (6) Å^3^, *Z* = 4, *T* = 100 K, Cu Kα radiation, λ = 1.54178 Å, *R*(*F*) = 0.023 [3506 reflections with *I* > 2σ(*I*)], *wR*(*F*^2^) = 0.060 (3578 reflections), Flack absolute structure parameter = –0.025 (6), CCDC deposition number 2337821. 

### Synthesis

**2,3-Dinitrothianthrene 6** 4,5-Difluoro-1,2-dinitrobenzene **5** (567 mg, 2.8 mmol) in EtOH (30 mL) was mixed with benzene-1,2-dithiol **4** (395 mg, 2.8 mmol) and Na_2_CO_3_ (4.0 g) then stirred at 75 °C for 20 h. The mixture was added to water (200 mL) and allowed to stand for 1 h. This was filtered and air dried for 2 days to give a bright yellow precipitate of the *title compound* (791 mg, 93%) as yellow crystals, 167–168 mp °C (from dichloromethane:light petroleum ether). λ_max_ (EtOH)/nm 243 (log ε 3.7) and 287 (3.5); ν_max_ (diamond) (cm^−1^) 3089w, 1532s, 1517s, 1442s, 1427s, 1349s, 1330s, 1238s, 899s, 851s, 748s, 661w, 444s and 423s; δ_H_ (400 MHz; D_6_DMSO) 7.45 (1H, d, *J* = 2.0), 7.46 (1H, d, *J* = 2.0); 7.63 (1H, d, *J* = 2.0), 7.65 (1H, d, *J* = 2.0) and 8.44 (2H, s); δ_C_ (100.1 MHz; D_6_DMSO) 125.3, 129.6, 129.9, 131.9, 141.4 and 142.3; *m/z* (Orbitrap ASAP) 306.9839 (M^+^ + H, 100%) C_12_H_6_N_2_O_4_S_2_H requires 306.9847. 

**2,3-Dinitrothianthrene-S-oxide 7** 2,3-Dinitrothianthrene **6** (500 mg, 1.63 mmol) in DCM (30 mL) was treated with *meta*-chloroperbenzoic acid (mcpba) (564 mg, 3.27 mmol) for 24 h at rt. The DCM layer was diluted with more DCM (70 mL), extracted with dilute KOH) (4 pellets of KOH dissolved in 200 mL of H_2_O), dried over MgSO_4_, then concentrated and purified by chromatography on silica. DCM eluted the *title compound* (191 mg, 37%) as a pale yellow powder, mp > 200 °C (from dichloromethane:light petroleum ether). λ_max_ (EtOH)/nm 226 (log ε 2.9) and 335 (2.1); ν_max_ (diamond) (cm^−1^) 3110w, 1530s, 1436s, 1356s, 1338s, 1069s, 1033s, 902s, 849s, 756s, 537s and 469s; δ_H_ (400 MHz; CDCl_3_) 7.67 (1H, t, *J* = 8.0 and 8.0), 7.79 (1H, t, *J* = 8.0 and 8.0), 7.91–7.94 (2H, m), 8.48 (1H, s) and 8.78 (1H, s); δ_C_ (100.1 MHz; CDCl_3_) 122.2, 125.3, 126.4, 126.7, 130.1, 130.5, 131.7, 136.6, 138.8, 141.3, 143.2 and 146.2; *m/z* (Orbitrap ASAP) 322.9879 (M^+^ + H, 100%) C_12_H_6_N_2_O_5_S_2_H requires 322.9796. 

**Thianthrene 9 and Thianthrene 10** Enantiomeric 2,3-dinitrothianthrene-S-oxide **7** (30 mg, 0.093 mmol) in EtOH (30 mL) and Et_3_N (28 mg, 0.28 mmol) were treated with (S)-phenylethylamine **8** (34 mg, 0.28 mmol) and heated under reflux for 24 h. After cooling the mixture was diluted with water (200 mL), treated with aqHCl (10 mL, 5M) and filtered giving a clear filtrate. The precipitate was dissolved in dichloromethane (100 mL), extracted with dilute aq HCl (30 mL, 1M) then water (100 mL), dried over MgSO_4_ and filtered. The two products were purified by chromatography on flash silica. They run close to each other on a TLC plate eluting with 10:90 ether/dichloromethane. The column was eluted with dichloromethane then 1:100 ether/dichloromethane eluted the *title compound* **9** (11 mg, 30%) as yellow crystals, mp 148–149 °C (from dichloromethane:light petroleum ether). λ_max_ (EtOH)/nm 264 (log ε 4.2), 334 (3.5) and 412 (3.5); ν_max_ (diamond) (cm^−1^) 3350w, 1597s, 1557s, 1474s, 1443s, 1418s, 1335s, 1278s, 1224s, 1115s, 1084s, 1033s, 967s, 908s, 836s, 753s, 699s, 535s and 466s; δ_H_ (400 MHz; CDCl_3_) 1.69 (3H, d, *J* = 7.0), 4.72 (1H, q, *J* = 7.0), 6.91 (1H, s), 7.311–7.42 (5H, m), 7.45 (1H, d, *J* = 8.0), 7.53 (1H, d, *J* = 8.0), 7.56 (1H, d, *J* = 8.0), 7.92 (1H, d, *J* = 8.0), 8.65 (1H, d, *J* = 8.0) and 8.71 (1H, s); δ_C_ (100.1 MHz; CDCl_3_) 24.8, 53.7, 114.0, 125.0, 125.5, 125.6, 126.9, 127.8, 127.9, 128.7, 129.1, 129.3, 130.5, 132.0, 137.6, 140.5, 142.2 and 144.8; *m/z* (Orbitrap ASAP) 397.0680 (M^+^ + H, 100%) C_20_H_16_N_2_O_3_S_2_ requires 397.0681. 1:100 Ether/dichloromethane eluted the *title compound* **10** (11 mg, 30%) as yellow crystals, mp 194–195 °C (from dichloromethane:light petroleum ether). λ_max_ (EtOH)/nm 265 (log ε 4.2), 334 (3.5) and 412 (3.5); ν_max_ (diamond) (cm^−1^) 3350w,1598s, 1558s, 1476s, 1279s, 1229s, 1112s, 1083s, 1032s, 969s, 910s, 832s, 753s, 703s, 598s, 534s and 464s; δ_H_ (400 MHz; CDCl_3_) 1.69 (3H, d, *J* = 7.0), 4.72 (1H, q, *J* = 7.0), 6.93 (1H, s), 7.32–7.45 (5H, m), 7.48 (1H, d, *J* = 8.0), 7.55–7.59 (2H, m), 7.93 (1H, d, *J* = 8.0), 8.64 (1H, d, *J* = 8.0) and 8.70 (1H, s); δ_C_ (100.1 MHz; CDCl_3_) 24.8, 53.7, 114.2, 124.9, 125.6, 127.1, 127.8, 127.9, 128.8, 129.0, 129.2, 130.5, 131.9, 137.6, 140.8, 142.4 and 144.8 (one peak is overlapping); *m/z* (Orbitrap ASAP) 397.0675 (M^+^ + H, 100%) C_20_H_16_N_2_O_3_S_2_ requires 397.0681. 

**2-Nitro-3-amino(phenylethyl)thianthrene 11** 2,3-Dinitrothianthrene **6** (100 mg, 0.33 mmol) in EtOH (30 mL) was treated with (S)-phenylethylamine **8** (80 mg, 0.65 mmol) and Et_3_N (66 mg, 0.65 mmol). The mixture was heated for 12 h. On cooling it was diluted with water and dilute aq HCl (20 mL, 1.0M) then extracted into DCM (100 mL). It was dried over MgSO_4_ and evaporated to dryness then purified by chromatography on flash silica. Elution with Et_2_O: DCM (2:98) eluted the *title compound* (33 mg, 27%) as an orange oil, λ_max_ (EtOH)/nm 257 (log ε 3.8), 348 (2.9) and 446 (3.0); ν_max_ (diamond) (cm^−1^) 3366w, 1599s, 1551s, 1506s, 1467s, 1446s, 1331s, 1273s, 1218s, 1088s, 1029s, 970s, 904s, 835s, 726s, 698s, 5644s, 470s and 444s; δ_H_ (400 MHz; CDCl_3_) 1.54 (3H, d, *J* = 7.0), 4.56 (1H, q, *J* = 7.0), 6.68 (1H, s), 7.07–7.14 (2H, m), 7.17 (1H, t, *J* = 8.0 and 8.0), 7.22–7.28 (5H, m), 7.32 (1H, d, *J* = 8.0), 8.14 (1H, s) and 8.32 (1H, d, *J* = 5.0); δ_C_ (100.1 MHz; CDCl_3_) 24.8, 53.2, 113.8, 120.6, 125.6, 125.7, 127.7, 127.8, 128.1, 128.7, 128.8, 129.2, 131.3, 133.7, 135.1, 142.9, 143.6 and 146.7; *m/z* (Orbitrap ASAP) 381.0741 (M^+^ + H, 100%) C_20_H_16_N_2_O_2_S_2_H requires 381.0732. 

**Thianthrene 9 and Thianthrene 10** 2-Nitro-3-amino(phenylethyl)thianthrene **11** (30 mg, 0.079 mmol) in DCM (30 mL) was treated with mcpba (27 mg, 0.16 mmol) at rt for 24 h. The mixture was extracted with dilute KOH (50 mL, 0.1M), washed with water (50 mL), dried over MgSO_4_, then purified by chromatography on flash silica. DCM eluted remaining starting material then 1% ether: DCM eluted firstly compound **9** (10 mg, 32%) of identical spectroscopic properties to that previously reported in this paper, followed by compound **10** (10 mg, 32%) of identical spectroscopic properties to that previously reported in this paper.

## 4. Conclusions

2,3-Dinitrothianthrene **6** was prepared by means of a novel one-pot condensation of benzenedithiol **4** with 4,5-difluoro-1,2-dinitrobenzene **5**. The activated fluorine atoms are displaced preferentially to a nitro group by the thiol groups. After oxidation of just one diarylthio ether to a sulfoxide, a nitro group conjugated to the sulfoxide and another nitro group was displaced by the chiral amine (S)-phenylethylamine **8,** chosen because of its availability and spatial difference. Two diastereomeric products **9** and **10** were produced, which ran back-to-back on a TLC plate eluting with dichloromethane/ether (98:2). These were separated using chromatography on flash silica and fully characterised. The absolute configuration of each diastereoisomer was established using X-ray crystal structure determinations. The more polar compound has a configuration **9** (*SR*) and the less polar spot has a configuration **10** (*SS*). These different configurations are apparent in the crystal structures as they flip the unsubstituted aryl ring in different orientations. The separation of the two diastereoisomers proves their stability as they are not interconverting and helps overcome concerns from others that sulfur-based chiral centres are not sufficiently stable for synthetic studies [23,24,25,26,27,28,29]. An alternative but similar pathway was developed to access thianthrenes **9** and **10** through the reaction of 2,3-dinitrothianthrene **6** with (*S*)-phenylethylamine **8** followed by the mcpba selective oxidation of the more electron-rich sulfur atom. Compound **11** was an oil, presumably because of its asymmetry, but with the conjugation of the amine to the sulfoxide, it became crystalline. The yield of sulfoxide formation was similar to that obtained by the mcpba oxidation of 2,3-dinitrothianthrene **6**. The sulfur oxidation yield is not influenced by an amine donor or a nitro group acceptor, but we did not observe the over-oxidation of the thianthrene ring to a sulfone or even a bis-sulfoxide, using two equivalents of mcpba. In summary, the crystal structures of these sulfoxides establish the absolute configuration of the chiral sulfur atom and prove the stability of the two diastereoisomers. 

## Data Availability

CCDC-2337820 and CCDC-2337821 contain the supplementary crystallographic data for this paper. These data can be obtained free of charge by emailing hello@ccdc.cam.ac.uk or by contacting The Cambridge Crystallography Centre, 12 Union Road, Cambridge, CB2 1EZ, UK; https://www.ccdc.cam.ac.uk/ (accessed on 5 March 2024).

## Supplementary Materials

The following supporting information can be downloaded at: https://www.mdpi.com/article/10.3390/ijms25084311/s1.
