# Supplementary material for: Chiral Thianthrenes
Source: Int J Mol Sci. 2024 Apr 13;25(8):4311. doi: 10.3390/ijms25084311 (PMC11050469; doi:10.3390/ijms25084311)
Supplement: Supplementary file 1 [file ijms-25-04311-s001.zip › ijms-2938668-supplementary.pdf]

Infrared Charts of Compounds 6-7, 9-11.

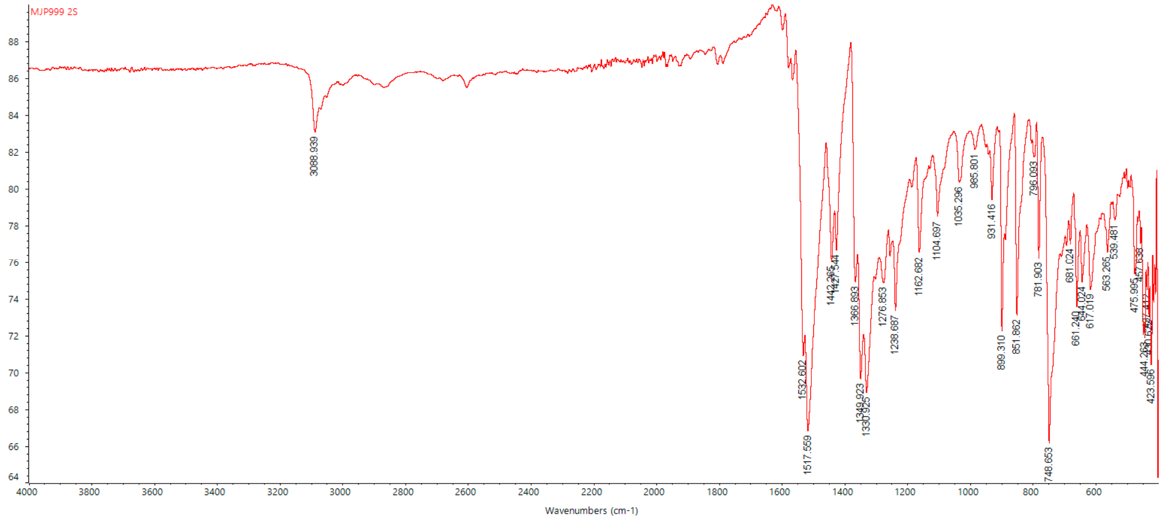

6

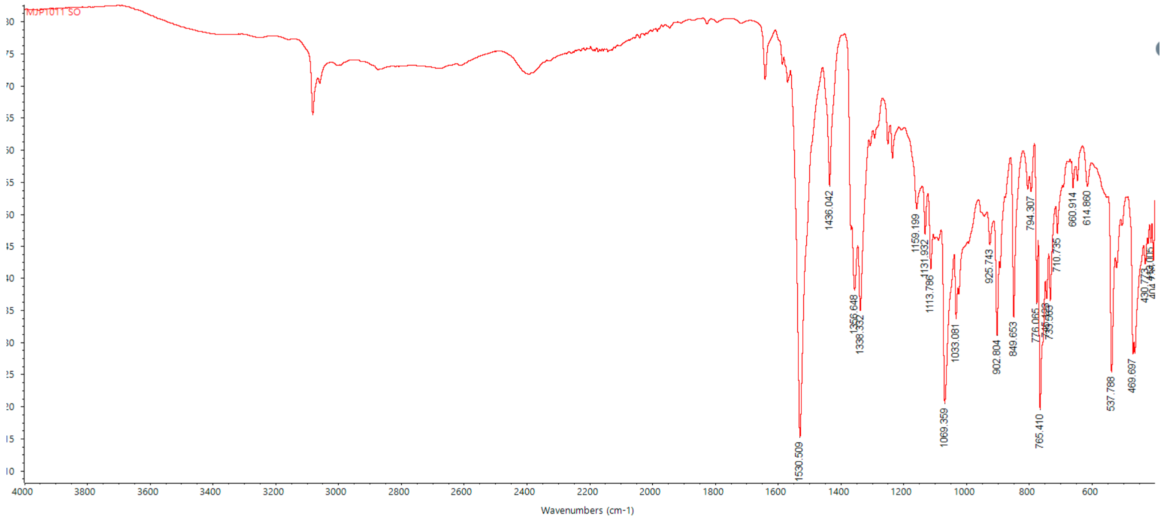

7

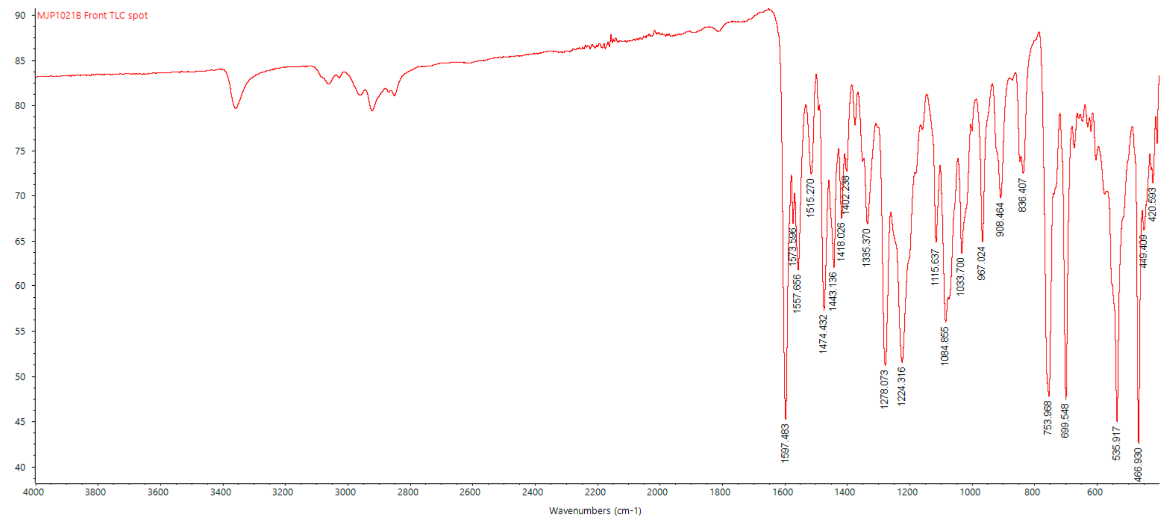
MJP1021B Front TLC spot
Wavenumbers (cm-1)
1597.483
1557.656
1573.595
1515.270
1474.432
1443.136
1418.026
1402.236
1335.370
1278.073
1224.316
1115.637
1084.855
1033.700
967.024
908.464
836.407
753.968
699.548
535.917
466.930
449.409
420.593

9

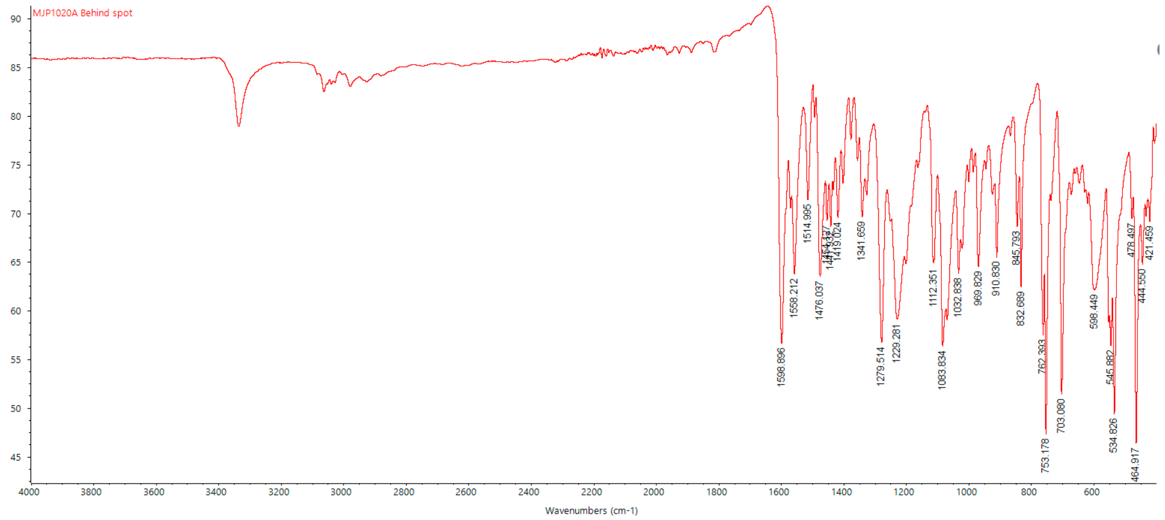
MJP1020A Behind spot
Wavenumbers (cm-1)
1598.896
1558.212
1514.995
1476.037
1419.024
1341.659
1279.514
1229.281
1112.351
1083.834
1032.838
969.829
910.830
845.793
832.689
762.393
753.178
703.080
598.449
545.882
534.826
478.497
464.917
444.550
421.459

10

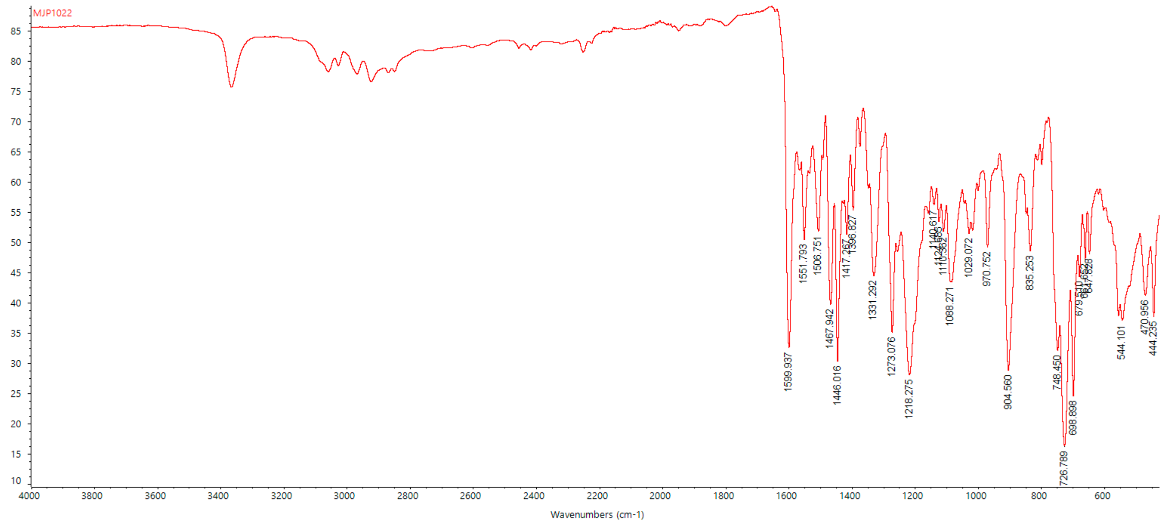
MJP1022
Wavenumbers (cm-1)
1599.937
1551.793
1506.751
1467.942
1446.016
1396.827
1331.292
1273.076
1218.275
1140.617
1088.271
1029.072
970.752
904.560
835.253
748.450
726.789
698.898
544.101
470.956
444.235

11

Proton and 13 Carbon NMR charts

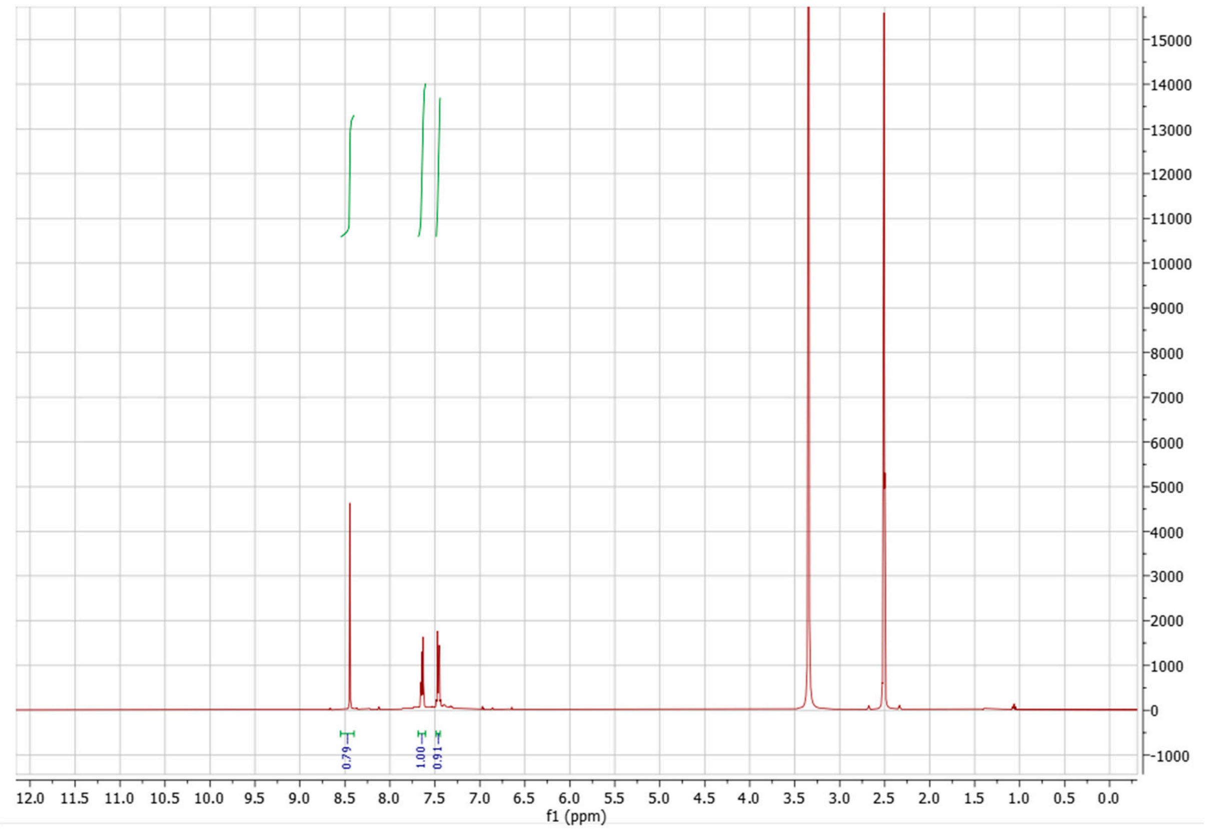

6 400 MHz Proton NMR D_6DMSO The peak at 2.5 ppm is DMSO and the peak at 3.4 ppm is water.

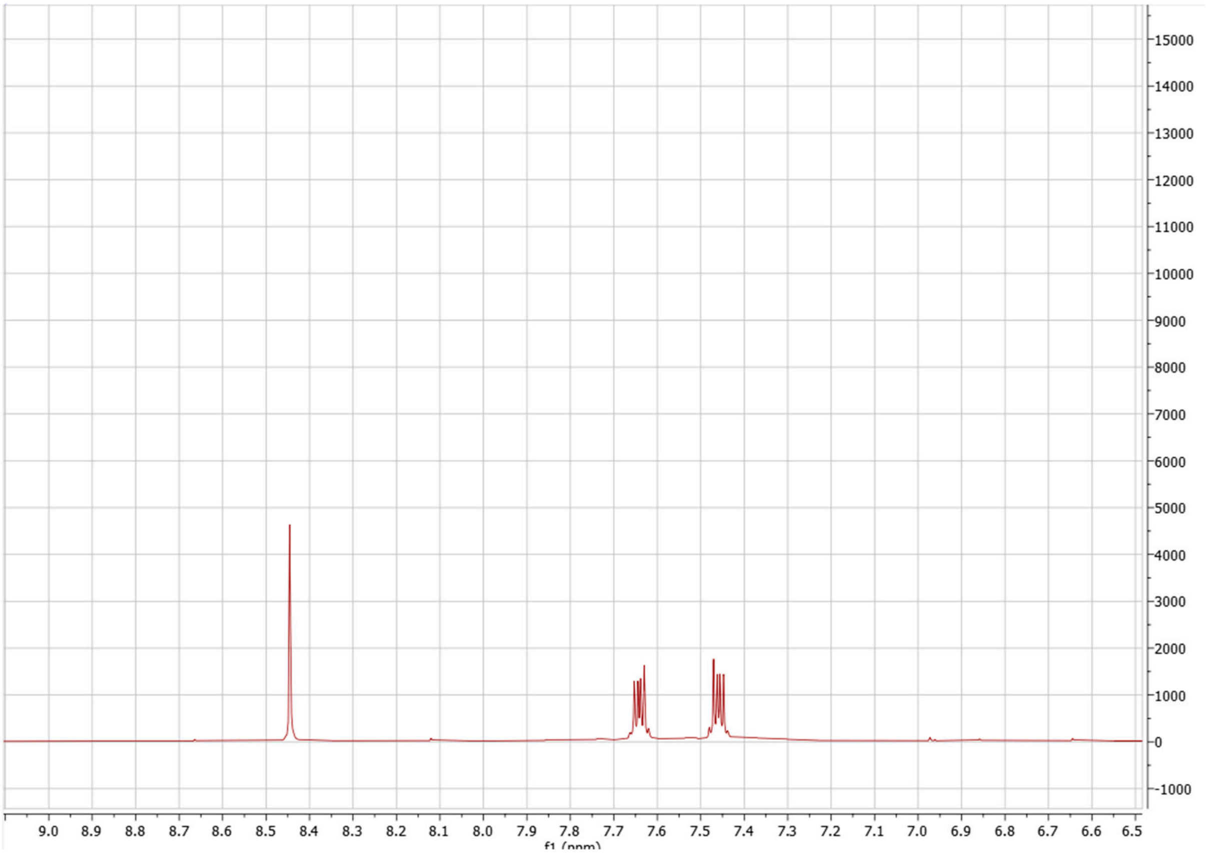

6 400 MHz Proton NMR D_6DMSO Expansion

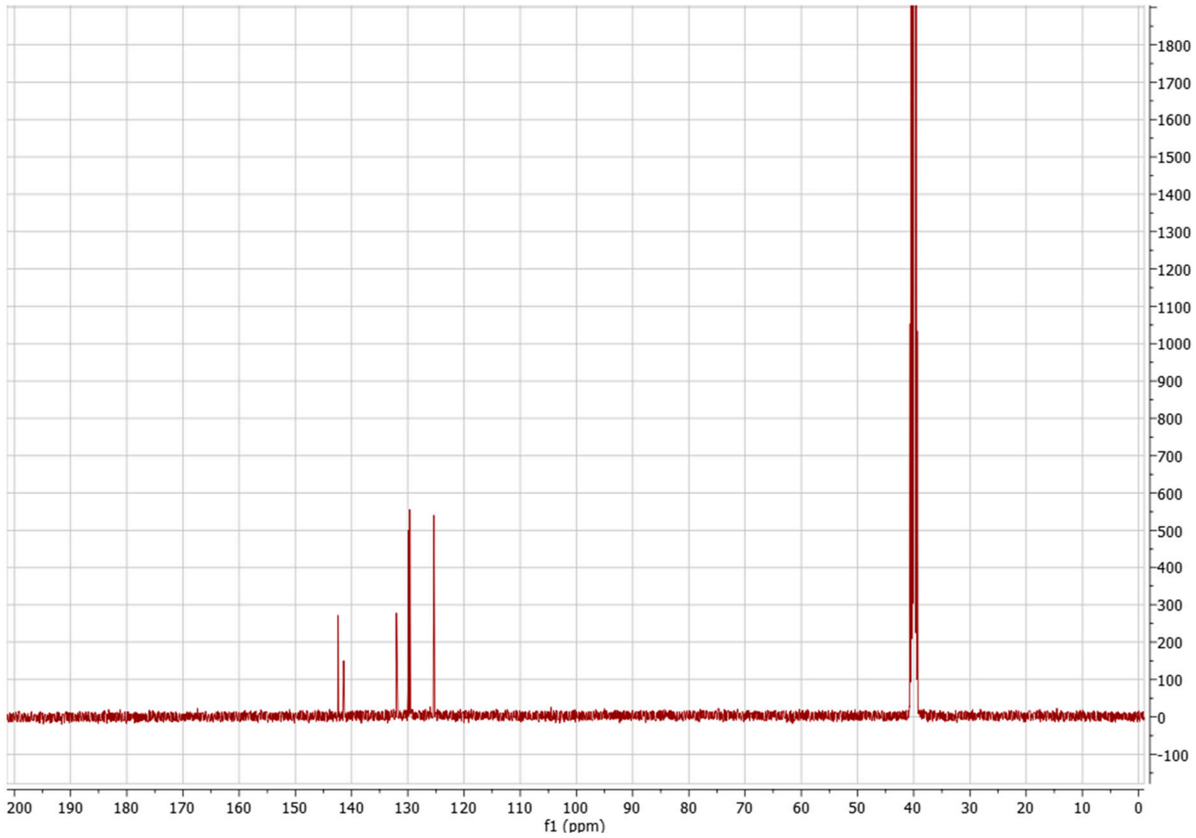

6 400 MHz 13 Carbon NMR D_6DMSO

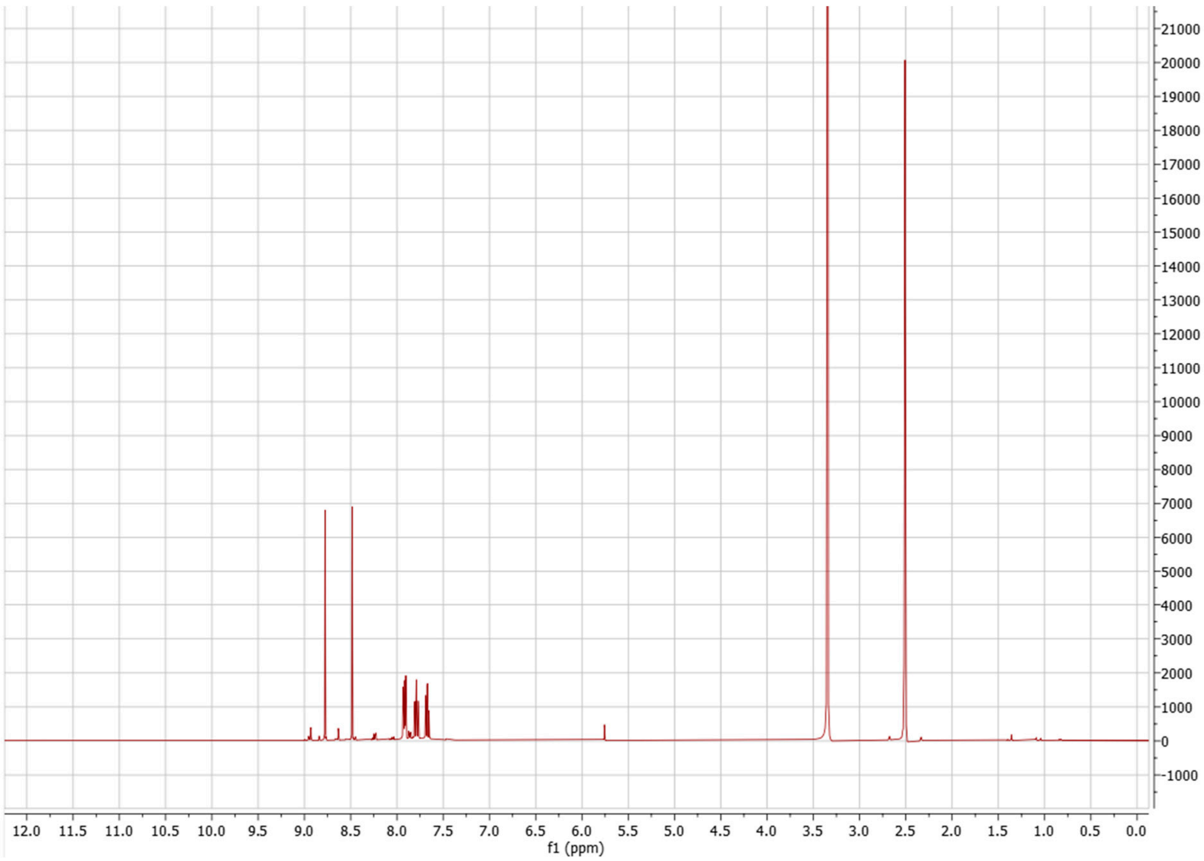

7 400 MHz Proton NMR D_6DMSO The peak at 2.5 ppm is DMSO and the peak at 3.4 ppm is water.

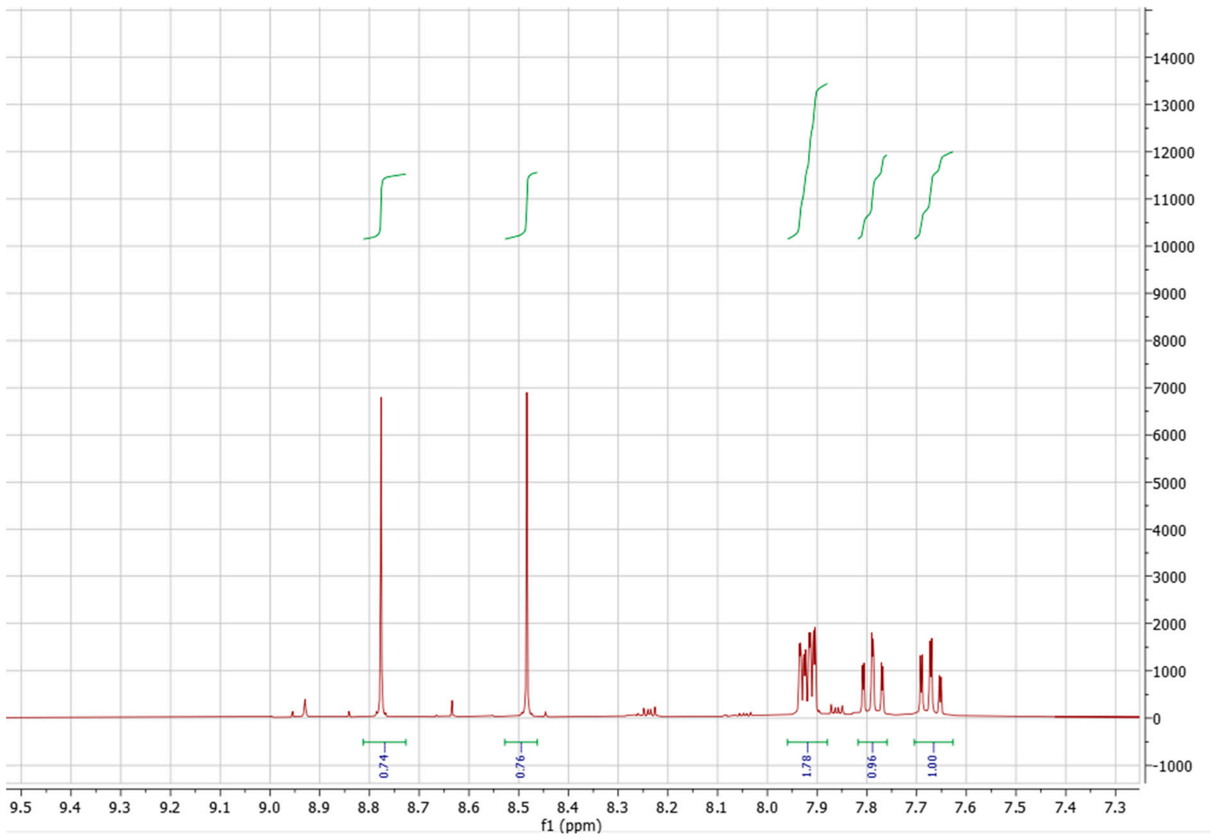

7 400 MHz Proton NMR D6DMSO Expansion

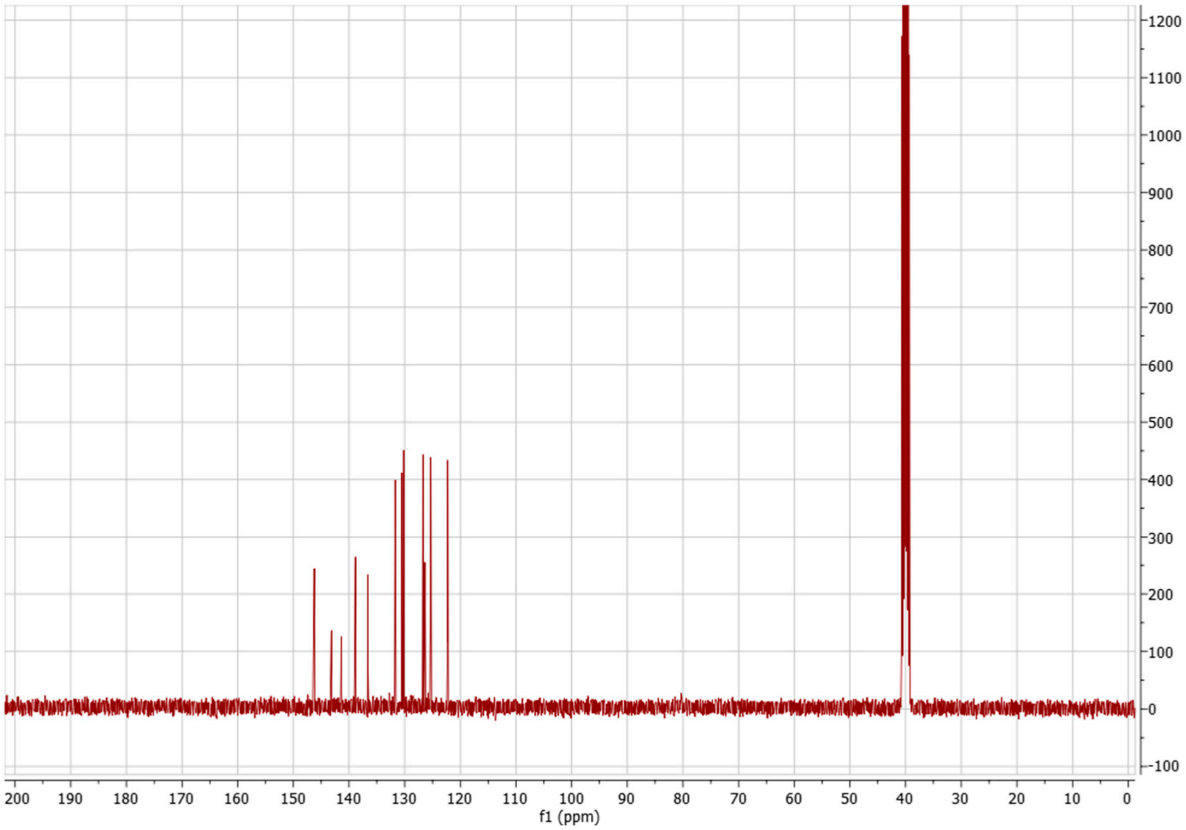

7 400 MHz 13 Carbon D_6DMSO NMR

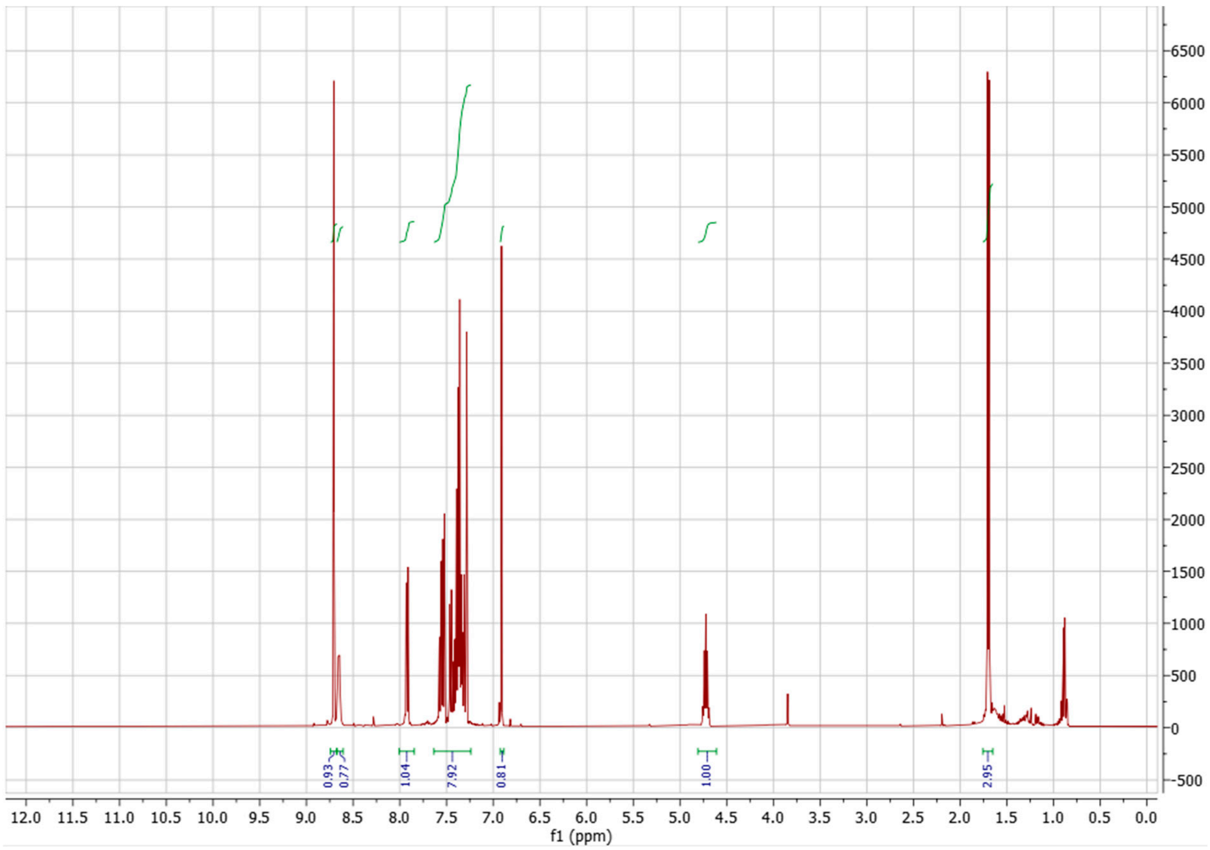

9 400 MHz Proton NMR $CDCl_3$

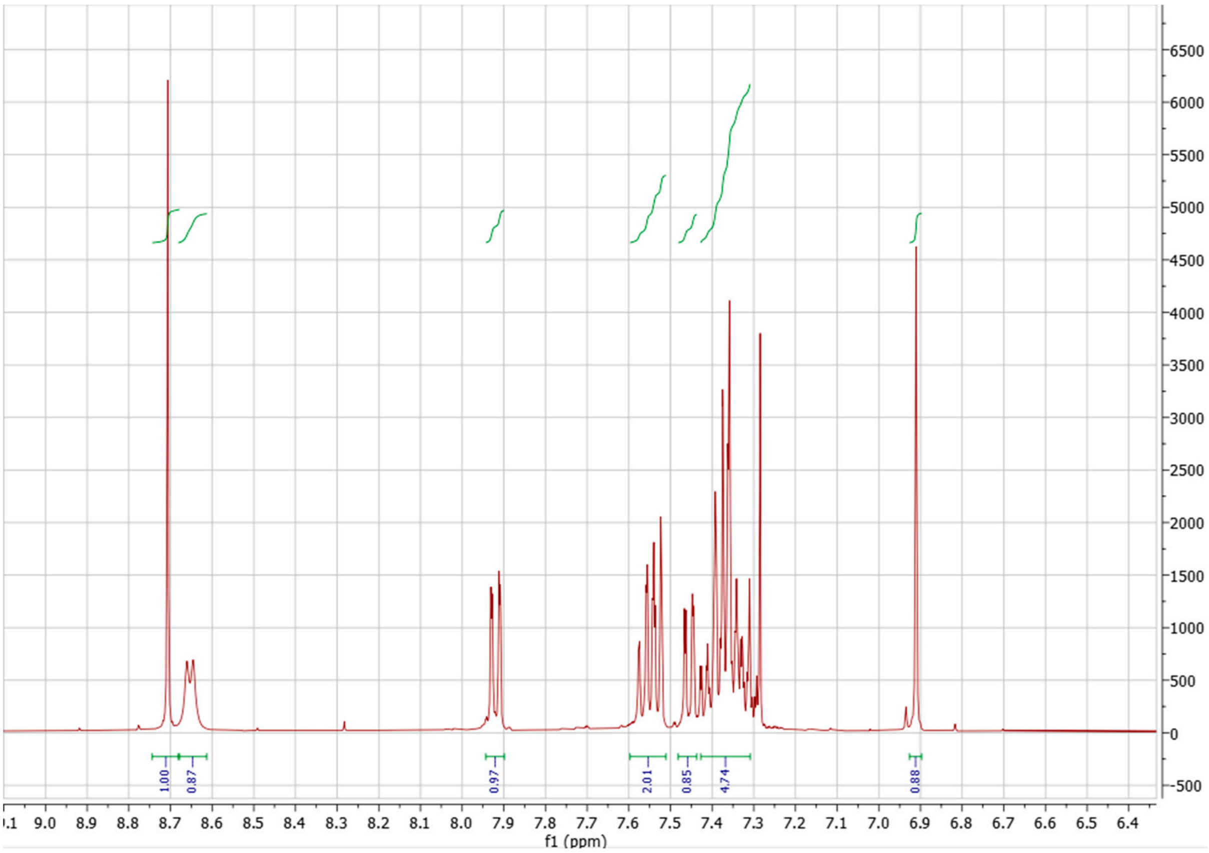

9 400 MHz Proton NMR $CDCl_3$ Expansion

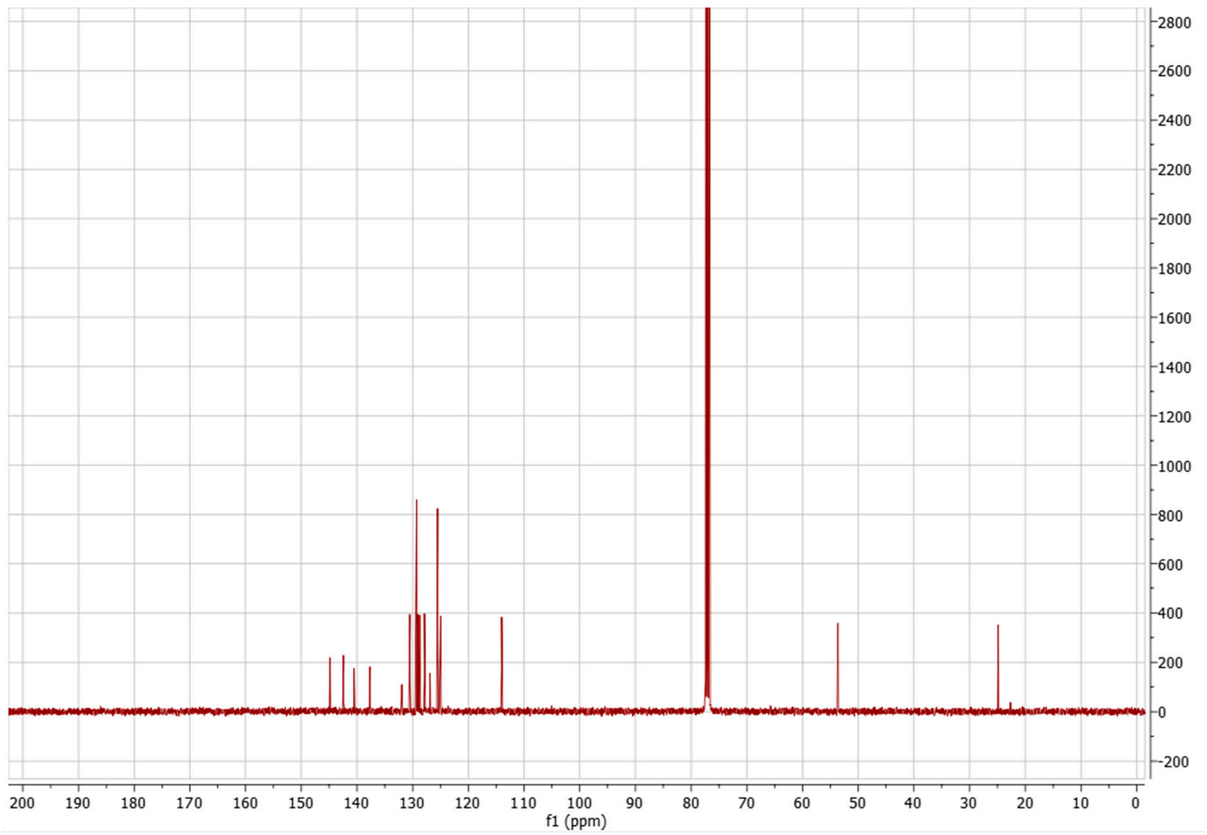

9 400 MHz 13 Carbon NMR $CDCl_3$

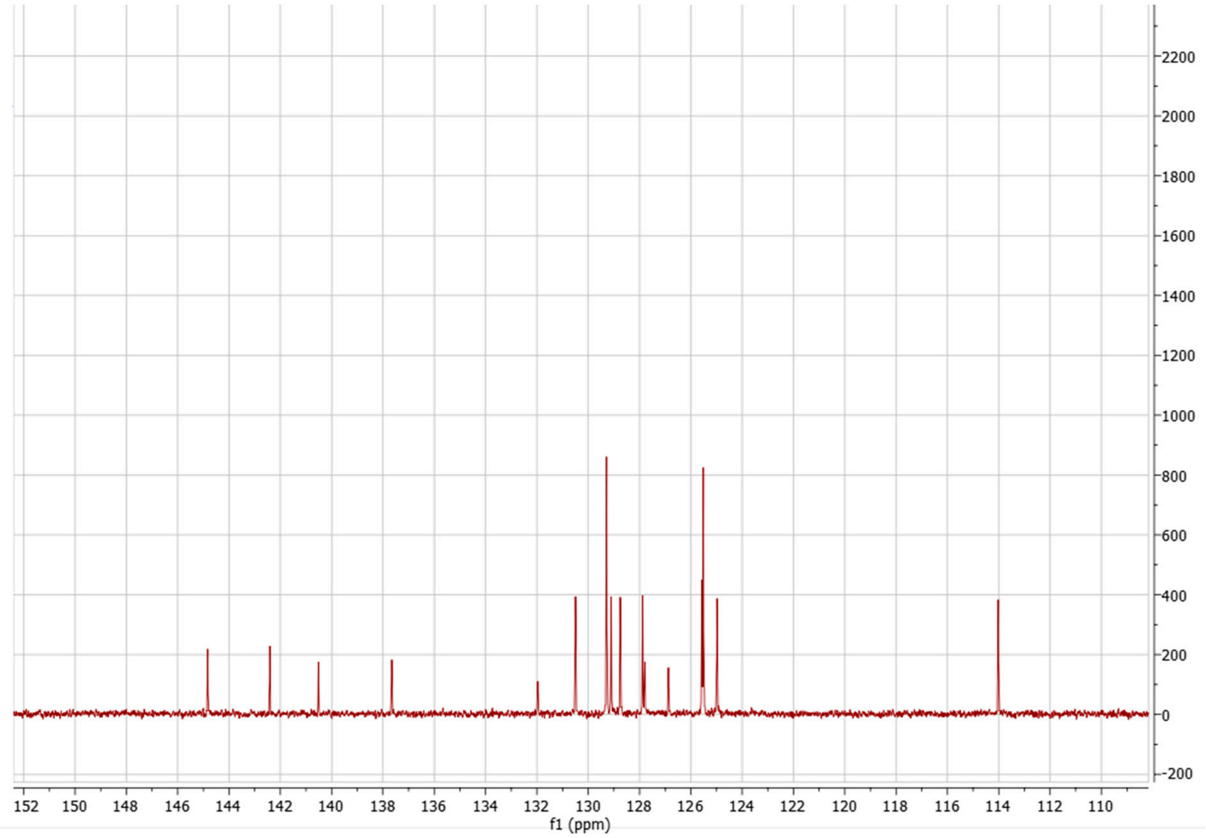

9 400 MHz 13 Carbon NMR $CDCl_3$ Expansion

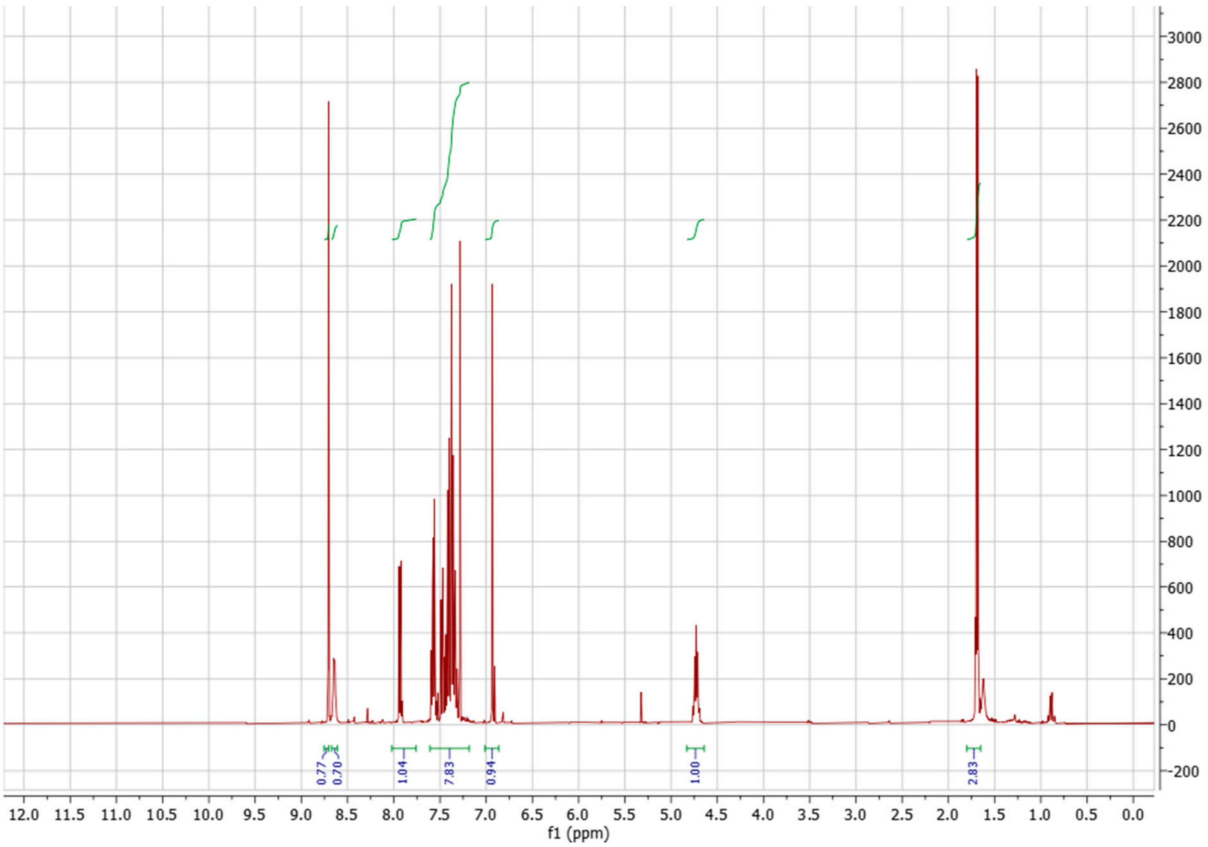

10 400 MHz Proton NMR $CDCl_3$

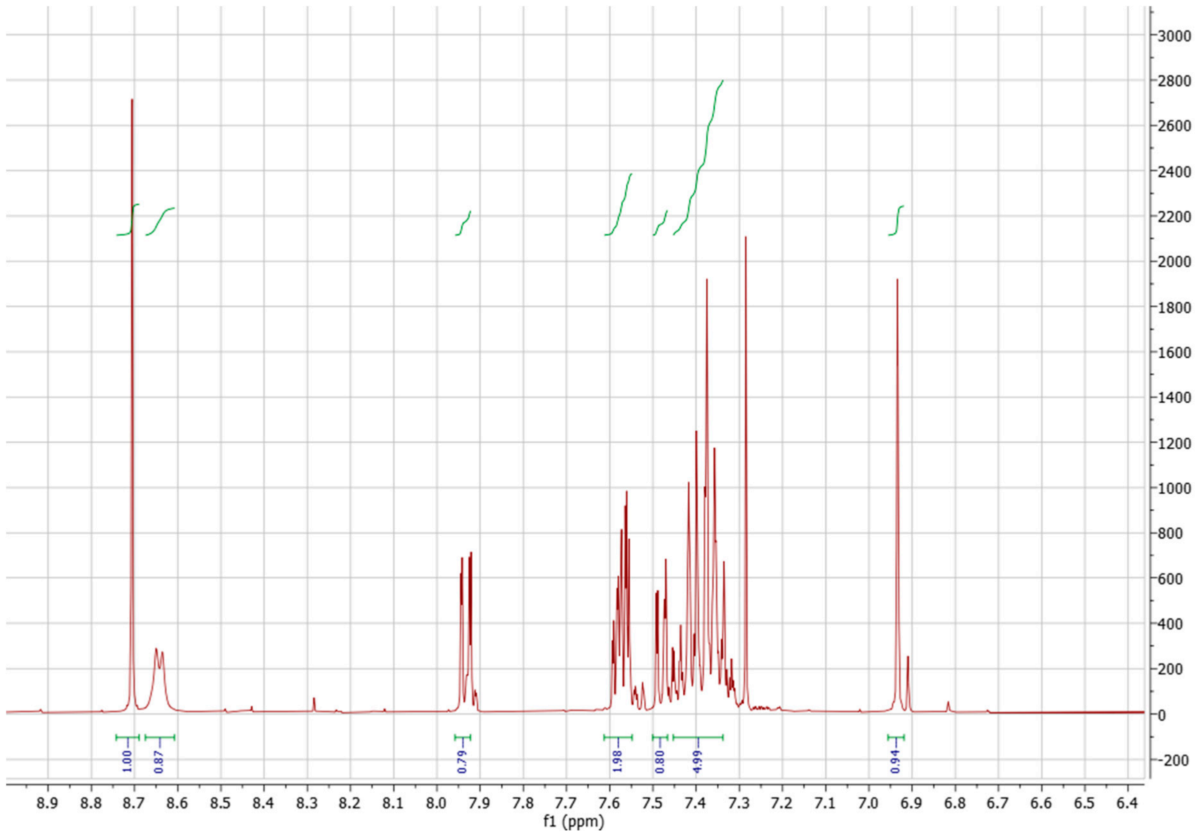

10 400 MHz Proton NMR $CDCl_3$ Expansion

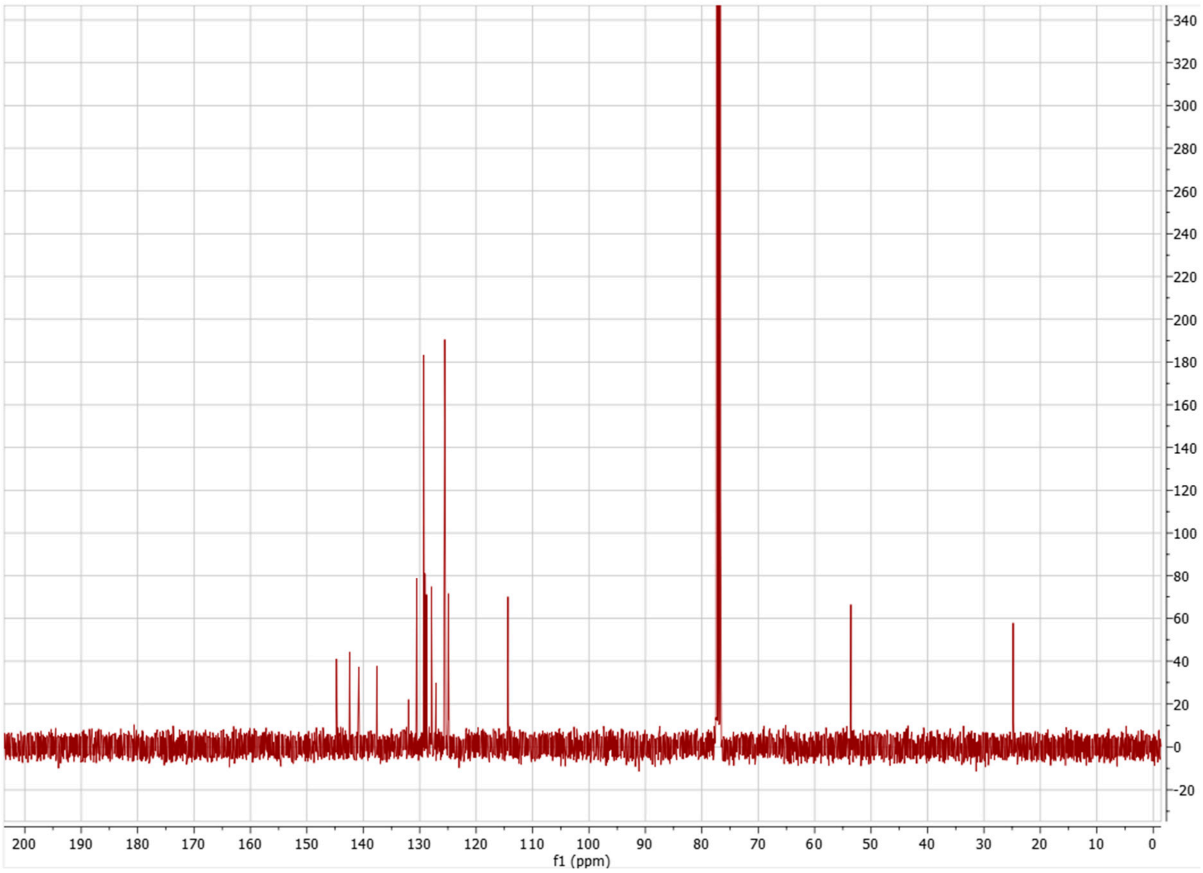

10 400 MHz 13 Carbon NMR $CDCl_3$

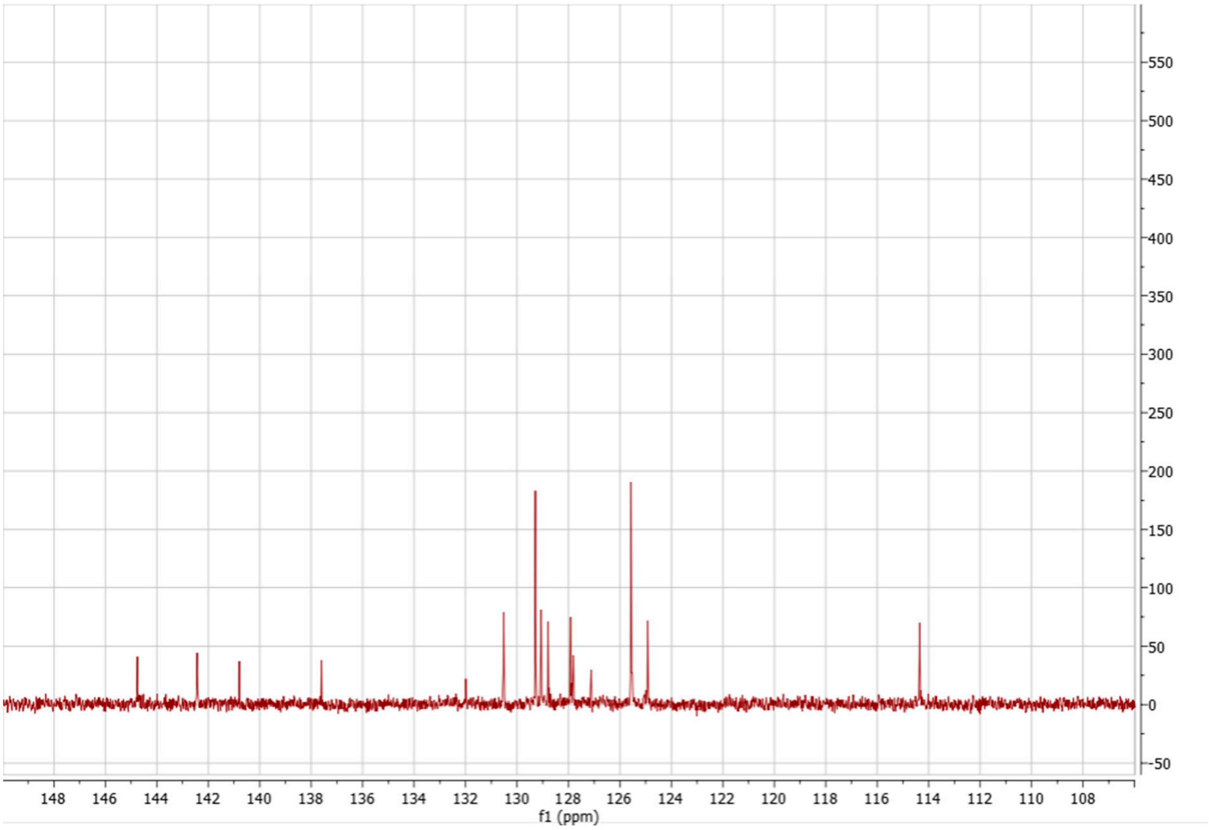

10 400 MHz 13 Carbon NMR $CDCl_3$ Expansion

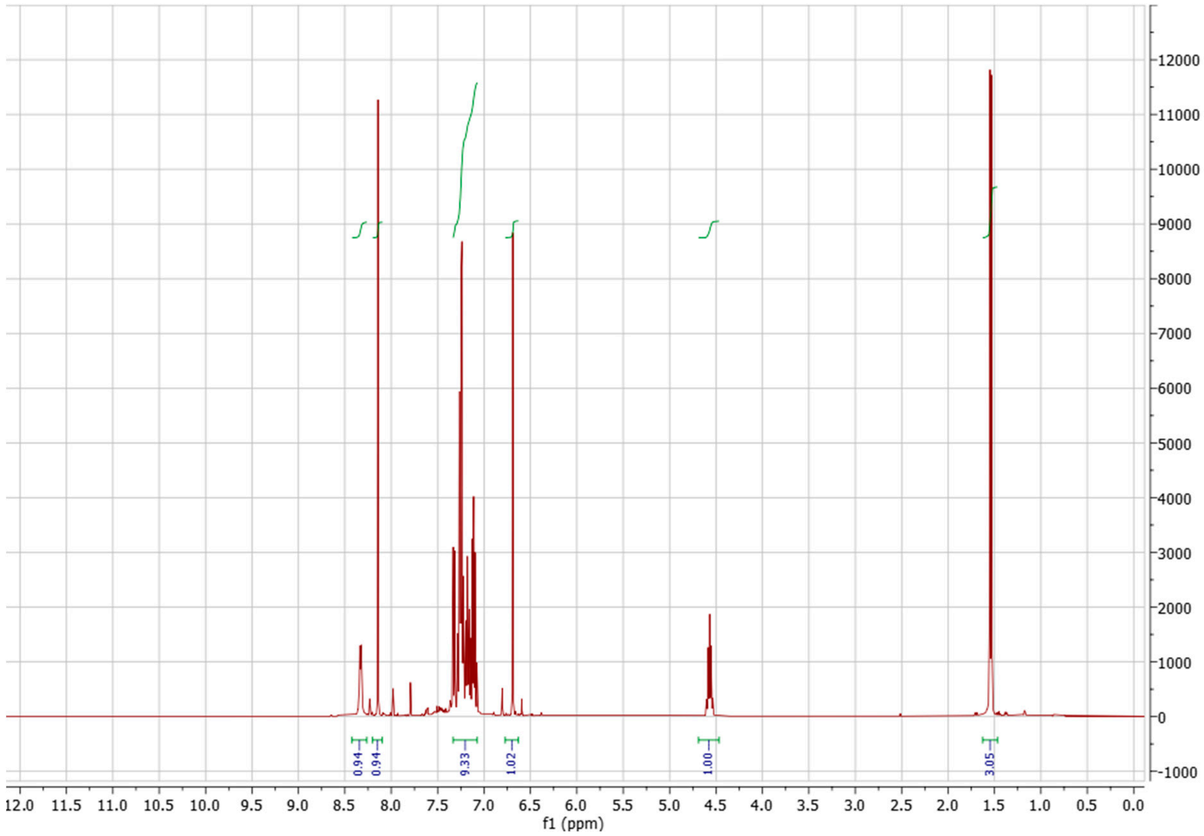

11 400 MHz Proton NMR $CDCl_3$

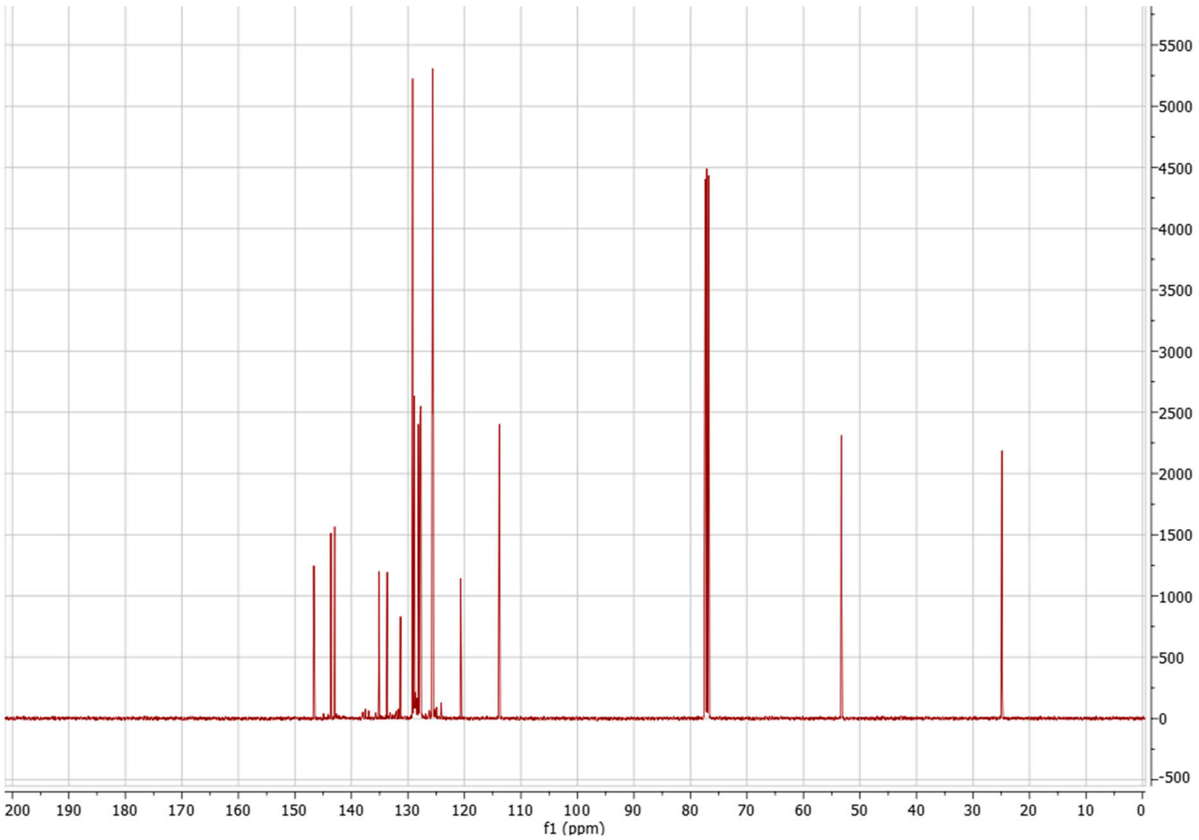

11 400 MHz 13 Carbon NMR $CDCl_3$

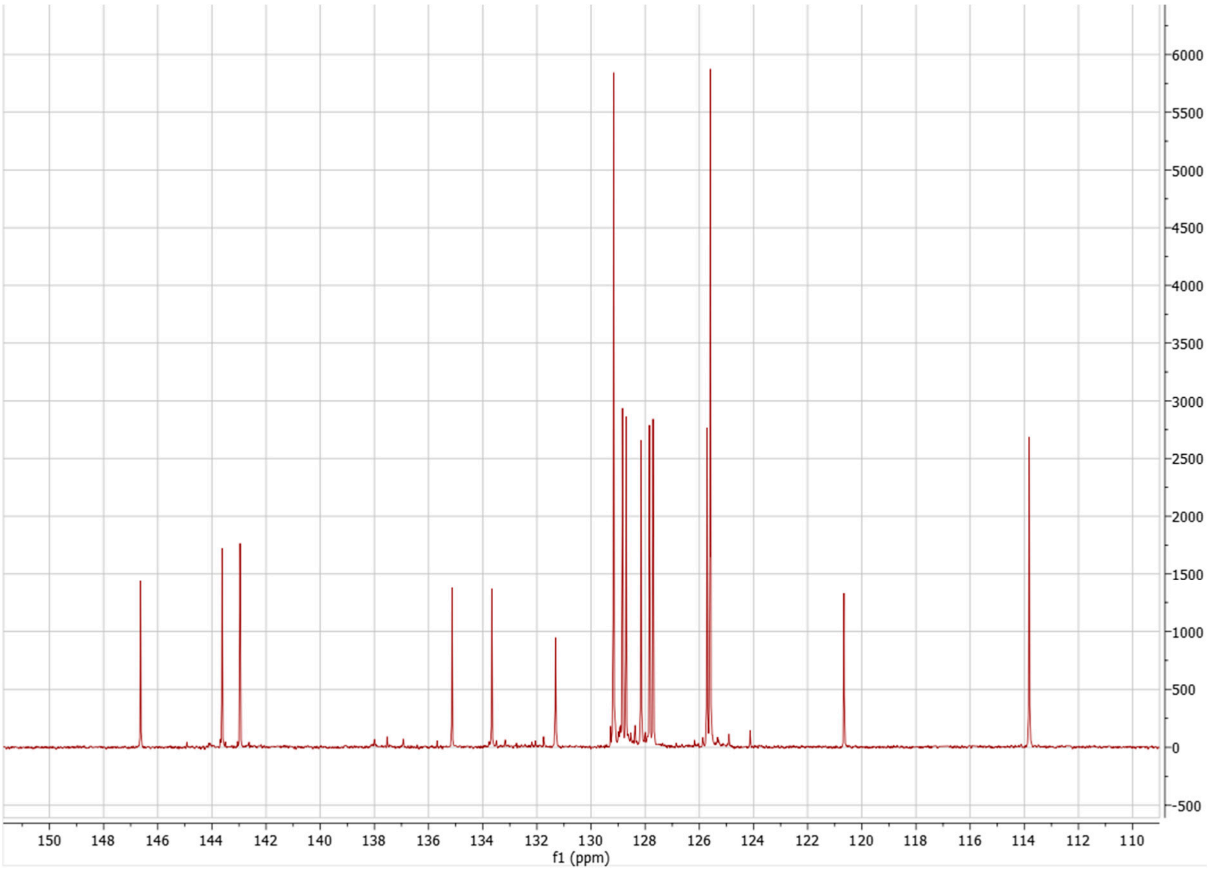

11 400 MHz 13 Carbon NMR $CDCl_3$ Expansion
